# Evaluation of prenatal calabash chalk geophagy on the developing brain of Wistar rats

**DOI:** 10.1016/j.ibneur.2024.03.007

**Published:** 2024-03-14

**Authors:** Moses B. Ekong, Anna Andrioli, Imaobong E. Israel, Edemeka I. Ifot, Samuel E. Dickson, Ilaria Scambi, Paolo F. Fabene, Giuseppe Bertini, Marina Bentivoglio

**Affiliations:** a Department of Anatomy, University of Uyo, Uyo, Nigeria; b Department of Neuroscience, Biomedicine and Movement Sciences, University of Verona, Verona, Italy; c Department of Engineering for innovation Medicine, University of Verona, Verona, Italy

**Keywords:** Calabash chalk, Neurotoxicity, Geophagy, Rat developing brain, Animal behavioural test

## Abstract

Calabash chalk (CaC) is an aluminium silicate hydroxide compound with heavy metal constituents, making it a potential neurotoxicant. Pregnant women often consume CaC as an antiemetic, which may interfere with the normal development of the foetal brain. Here, we evaluated the effects of CaC administration in pregnant rats on the brain of the offspring. Wistar rat dams were assigned to one of three groups: control, 200 mg/kg and 800 mg/kg of a CaC suspension. Administrations lasted 14 days (gestation days 7–20). On day 14, 5-bromo-2′-deoxyuridine (BrdU) was administered and dams were allowed to term. Behavioural tests were performed on different days as the pups matured, and they were sacrificed on post-natal days 30 and 60. Brains were processed for histology and Western blotting. Results showed no significant differences in surface righting reflex, cliff avoidance, negative geotaxis and open-field activity. No hippocampal and somatosensory cortical cytoarchitectonic alterations and no significant signs of glial fibrillary acidic protein (GFAP) activation were observed. Neuronal nuclei counts showed variability in the somatosensory cortex and hippocampus of the CaC group. BrdU-positive cells were significantly lower in the 200 mg/kg group and higher in the 800 mg/kg group. Doublecortin-X-positive cells were not different in all the CaC groups. Astrocytes and microglia Western blotting quantification confirmed no significant increase in pup glial cells in adulthood. Prenatal consumption of CaC at indicated dosages may not be deleterious to the developing brain, especially after cessation of exposure and during maturation of the animal. However, the differences in neuronal and glial populations may be due to their ability to cope with CaC.

## 1 Introduction

**Fig. 1:**
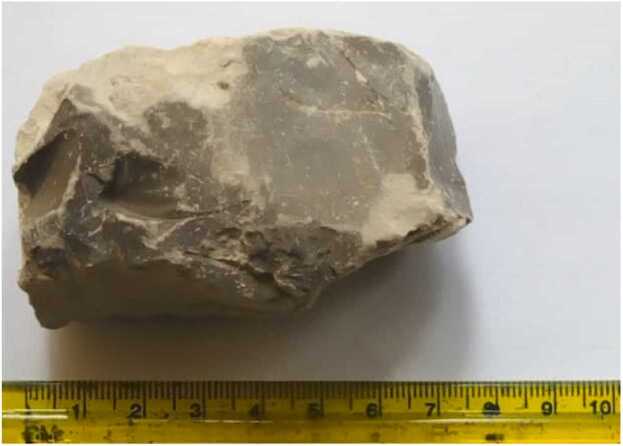
Calabash chalk block sample used in the experiment.

Geophagy, the practice of intentionally eating earth and soil-like substances, is common in many tribal societies, and can be described as a craving for non-food substances associated with pleasure (GUNDACKER et al., 2017, HUEBL et al., 2016, ROY et al., 2018). This practice, also known as *pica*, cuts across sex and social strata (Kariuki et al., 2016). One of these substances is the calabash chalk (Fig. 1), commercially available and consumed mainly by pregnant and post-partum women as an antiemetic, but also for its pleasant or soothing taste, to the point of becoming addictive (AKPANTAH et al., 2010, Ekong et al., 2008).

Calabash chalk (CaC) is a mixture of clay and chalk that occurs naturally but can be formulated artificially, where salt (sodium chloride) is sometimes added. It is widely available in many developing countries, particularly Nigeria and other sub-Saharan African countries, but its consumption has also been reported in developed countries (AKPANTAH et al., 2010, EKONG et al., 2012b, Ekong et al., 2008, ROY et al., 2018). It has an aluminium silicate hydroxide structure, which belongs to the group of kaolin clays, with the formula: Al_2_ Si_2_ O_5_ OH_4_ (Dean et al., 2004)_._ Other constituents include metals, metalloids, and persistent organic pollutants (DEAN et al., 2004, EKANEM et al., 2015, EKONG et al., 2015b). The chalk also contains lead, vanadium, chromium, and arsenic in considerable quantities (DEAN et al., 2004, EKANEM et al., 2015, EKONG et al., 2015b, ZABIN, 2017). Some of these metals, including lead, vanadium and aluminium, have been reported to cross the placental barrier (JAISHANKAR et al., 2014, MANDUCA et al., 2017, RAHBAR et al., 2015, REHMAN et al., 2018), thus having unlimited access to the foetus. On the other hand, persistent organic pollutants have been reported to be transferable from mother to foetus, implying their ability to cross the placental barrier (PORPORA et al., 2013, RIBEIRO et al., 2017, VIZCAINO et al., 2014). Altogether, these CaC components can be detrimental to the foetus, as they accumulate in the tissues (Ekong et al., 2012a) including the brain, since at this stage the blood-brain-barrier is still immature and allows exogenous substances to cross easily (Saunders et al., 2012).

Some CaC constituents are known neurotoxins, capable of inducing mental retardation in both children and adults (LIU and LEWIS, 2014, PERLROTH and CASTELO BRANCO, 2017), which adds to the concern that their accumulation in the developing brain may be detrimental. Indeed, preliminary experimental reports on maternal and foetal brain animal models have shown proliferation of cerebral cortical cells, hypertrophy of pyramidal cells, chromatolysis and vacuolations (EKANEM et al., 2015, EKONG et al., 2015a). These findings show the adverse effect of CaC on the developing brain. However, there are still substantial gaps in knowledge on the effects of CaC ingestion, especially on other stages of brain development. Therefore, the present study investigated the impact of maternal prenatal consumption of CaC on the maturing brain of rat pups.

## 2 Material and methods

### 2.1 Animals handling

Eighteen pregnant Wistar rat dams were randomly grouped in cages of three dams per cage and were divided equally (n = 6) into three experimental groups: control, 200 mg/kg, and 800 mg/kg of the CaC suspension. To obtain pregnancy, sexually naïve, but mature female Wistar rats, 3 months of age, (at proestrous) of body weight 170–180 g and same strain males of body weight 200–210 g, were caged overnight. The dams were allowed to mate within these cages along with others. The presence of seminal plugs the following day indicated mating and was designated gestational day 0. The dams were allowed to term before being transferred to their individual cages with their pups. All the animals were kept at room temperature of 27–30℃ in 12:12-hours light and dark cycles in the animal facility of the Faculty of Basic Medical Sciences, University of Uyo. Ethical permission for the research was obtained from the Ethical Committee of the Faculty of Basic Medical Sciences of the University of Uyo in Nigeria (FBMS27/01/2016), while the rats were treated in accordance with European Communities Council Directive (86/609/EEC) on the care and use of animals for scientific purposes. All efforts were made to minimize the number of animals used and their suffering.

### 2.2 Preparations and administrations of CaC and 5-bromo-2′-deoxyuridine

Different CaC blocks were obtained from a local market in Calabar, Nigeria. CaC samples were prepared by dissolving the powdered form in water, to obtain a 40 mg/mL suspension. Since CaC is only partially miscible in water, the chalky suspension was stirred before administration. Concentrations of 200 mg/kg (Ekong et al., 2015a) and 800 mg/kg (four times of 200 mg/kg) of the CaC suspension were administered orally by gavage, over a period of 14 days (gestation days 7–20). Two additional samples (in block and powdered forms) were later acquired (not at the same time since the original samples were exhausted) from the same vendor and sent to the University of Verona, Italy, for inductively coupled plasma mass spectrometry (ICP-MS) analysis. Meanwhile, 5-bromo-2′-deoxyuridine (5-BrdU, 250 g, Tocris Bioscience™ 5015/50) was dissolved in distilled water and the pH adjusted to 7.35. The BrdU solution was then filtered and stored at 4°C before administration. Immediately after the last administration of the chalk solution, dams were administered BrdU intraperitoneally (i.p., 60 mg/kg). The eighteen dams were allowed to term, and their pups were weighed at birth and weekly thereafter. Six pups (male and females) were culled per group, making a total of eighteen.

### 2.3 ICP-MS analysis of CaC

CaC powdered samples were treated with microwave-assisted digestion in *aqua regia* (67% HNO_3_ and 33% HCl) using the procedure according to Al-Rmalli et al. (2010). Briefly, 50 mg of each sample were added to 5 mL of *aqua regia* and left overnight at room temperature. Samples were then digested for three hours in a microwave and subsequently diluted with pure water to a final HNO_3_ concentration of approximately 1%. The solution was centrifuged for 15 minutes at 5000 rpm, and the supernatant was collected for analysis. Elements of interest were determined by ICP-MS using an iCAP™ RQ ICP-MS (Thermo Scientific). To correct for instrumental drifts and plasma fluctuations, the solutions were spiked with internal standard elements (10 ppm each): Scandium (Sc), Germanium (Ge), Yttrium (Y), and Indium (In). The mean concentration value and standard deviation (SD) were calculated for each element.

### 2.4 Behavioural testing

Developmental, motor, and emotional behavioural tests were performed after birth on the pups (FEATHER-SCHUSSLER and FERGUSON, 2016, HEYSER, 2004). On post-natal day (PND) 7, 8, and 10, respectively, surface righting reflex (ability to return to standing on its feet from a supine position), cliff avoidance (ability to turn away from a potential fall off a cliff), and negative geotaxis (ability to turn to a position away from gravity) tests were carried out. Each pup was allowed three trials per day. The surface righting reflex and cliff avoidance tests were repeated on PND 14 and 15, respectively. On PND 25 the open field test (including line crossing, rearing, and grooming evaluation) was performed.

### 2.5 Animal sacrifice

On PND 30 or 60, the rats were anaesthetized with 50 mg/kg body weight ketamine hydrochloride (i.p.) and sacrificed by perfusion fixation using 4% paraformaldehyde in phosphate-buffered saline (PBS). The whole brain was removed and post-fixed in 4% paraformaldehyde for 48 hours. Brains were cryoprotected in 30% sucrose and 40 µm serial coronal sections were obtained with the freezing microtome (˗22 °C).

### 2.6 Histological and immunohistochemical procedures

Representative serial sections of the somatosensory cortex and dorsal hippocampus from each animal (six brain sections per animal) were processed for Nissl staining using Cresyl violet stain, and for myelin impregnation using Black-Gold II. Sections for Black-Gold histochemistry were processed following Schmued and Slikker (1999). Briefly, brain sections in PBS were rinsed in distilled water and transferred to 0.3% Black-Gold II (Histo-Chem, Jefferson, AR, USA) in distilled water in the oven at 60℃ for 10 min. After rinsing, slides were transferred into 1% sodium thiosulphate solution for three minutes; then, rinsed in running tap water, dehydrated, cleared and cover slipped with Entellan (Merck, Darmstadt, Germany).

Free-floating sections (six per animal for each antibody) were processed and immunolabelled for glial fibrillary acidic protein (GFAP), neuronal nuclei (NeuN), Doublecortin (DCX) and BrdU. Briefly, sections were pre-treated with 1% hydrogen peroxide for 15 minutes, and subsequently incubated in 5% normal serum and 0.3% Triton-X-100 in PBS for 1 hour. The sections were then incubated in one of the following primary antibodies: polyclonal rabbit anti GFAP (1:500, Dako, Glostrup, Denmark; 20013971), mouse anti NeuN (1:500; EMD Millipore, Temecula; 2683919), goat polyclonal anti DCX (1:200; Santa Cruz Biotechnology, CA; L0209), overnight at room temperature. These were followed by repeated washings and by 2 hours of incubation in their respective appropriate biotinylated secondary antibodies (1:200, Vector Labs. Burlingame, CA). After washings, sections were incubated for 90 minutes in avidin-biotin complex (ABC kit, Vectastain, Vector Labs). Detection of the reaction was performed using 3’-3’-diaminobenzidine as chromogen. The sections were then dehydrated, cleared and cover-slipped with Entellan.

BrdU sections were treated with 1% hydrogen peroxide for 15 minutes and were then incubated in normal hydrochloric acid for 2 hours before treatment with 5% normal serum for 1 hour. They were subsequently incubated overnight in monoclonal mouse anti BrdU (1:200, Dako, 20007454), followed by 2 hours of incubation in biotinylated anti-mouse IgG (1:200, Vector Labs) and 90 minutes in ABC complex. Detection of reaction was achieved with 3’-3’-diaminobenzidine as chromogen. The sections were dehydrated, cleared and cover-slipped with Entellan.

Sections on slides were observed under a microscope and quantified by stereological cell counting using Stereoinvestigator software (MBF Bioscience, Williston, VT, USA).

### 2.7 Western blot

Microglia-specific proteins (CD11b and Iba1) and astrocytic cell protein (GFAP) were analysed in the cortex and hippocampus on controls and on the 800 mg/kg animals, sacrificed at days 30 and 60 (n= 4 per group), using a previously validated technique (AZEEZ et al., 2016, TANCA et al., 2015). From the cut series, two brain sections per animal containing the somatosensory cortex and the dorsal hippocampus were chosen, resected and pooled.

For protein extraction, collected tissues were processed with Qproteome FFPE Tissue Kit (Qiagen, Hilden, Germany), according to the manufacturer’s instructions. Protein concentration was determined using the Pierce™ Detergent Compatible Bradford Assay Kit (23236, Thermo Fisher Scientific, Rockford, USA), and 25 μg of protein extraction was used for western blotting.

Proteins were resolved by 4–20% SDS-polyacrylamide gel (Biorad, Labs, Hercules, CA) and transferred to polyvinylidene difluoride (PVDF) membrane*.* Blotting membranes were cut at 75 kDa and at 25 kDa for the immunoblotting of anti-CD11b (127 kDa), anti-GFAP (50 kDa), anti-Iba1 (16,7 kDa) and actin protein (housekeeping 35 kDa).

Membranes were preincubated in 5% non-fat dry milk dissolved in 0.05% PBS-Tween for 90 min on a shaker. The membranes were then incubated for detection respectively with 1:500 mouse anti-CD11b (AbD Serotec, UK), 1:500 mouse anti-GFAP antibody (ab11427, Abcam, Cambridge, UK), 1:500 rabbit anti-Iba1 (019–19741; Wako Chemicals, USA), and 1:1000 anti-β-actin (ab6276, Abcam, Cambridge, UK), overnight at 4°C. After repeated washings, appropriate HRP-conjugated secondary antibodies (all from DAKO, Glostrup, Denmark) 1:5000 were used for visualization. Chemiluminescent reagent (ECL, Immobilion Forte, WBLUF0100, Millipore, Burlington, MA) was used to expose the films and detected with G:BOX F3 GeneSys (Syngene, Cambridge, United Kingdom).

### 2.8 Statistical analysis

Data from behavioural tests and cell counts were analysed with analysis of variance (ANOVA) using GraphPad Prism (version 5) and Tukey post-hoc. Statistically significant data were determined at a probability level of p ≤ 0.05 and data represented as mean ± standard error of the mean (SEM).

Densitometric analysis of Western blot was performed with the open source ImageJ software (Schneider et al., 2012). Data were analysed with SPSS 2 software, using two-way ANOVA for mean analysis and Bonferroni post-hoc test. Values were expressed as normalization of proteins’ optical density relative to internal loading control (β-actin).

## 3 Results

### 3.1 CaC analysis

CaC analysis showed the presence of heavy metals and arsenic, a metalloid. Aluminium had the highest concentration with cadmium being the lowest. Iron and potassium concentrations were equally high. A comparison with a previous study showed similar trend of concentration (Table 1).

**Table 1:** ICP-MS analysis of CaC.

Elements analysed	Concentration of elements (mg/Kg)	Concentration of elements (mg/Kg) CaC Ekong et al. (2015b)
Aluminium (Al)	38726±18257	160000
Potassium (K)	6385±2123	5500
Calcium (Ca)	943±190	160
Vanadium (V)	50±13	125
Chromine (Cr)	43±12	130
Manganese (Mn)	31±39	40
Iron (Fe)	8477±7105	15000
Cobalt (Co)	30±37	4,1
Nickel (Ni)	29±17	25,5
Copper (Cu)	23±16	15,5
Zinc (Zn)	19±17	<100
Arsenic (As)	9,4±1	11,5
Cadmium (Cd)	0,15±0,22	0,76
Lead (Pb)	36,8±5,9	57

Results are presented as Mean±SD.

### 3.2 Pup body weight monitoring

**Fig. 2:**
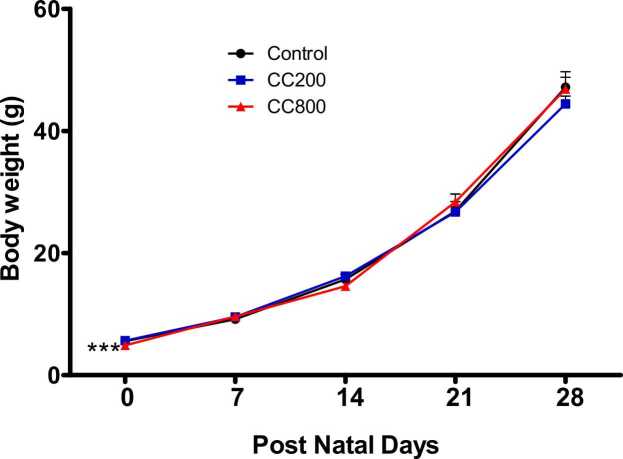
Weekly body weight of the pups from birth. No differences in body weight were observed, at p < 0.05, except at birth (day 0). CC200 indicates 200 mg/kg CaC group; CC800 indicates 800 mg/kg CaC group. *** Significantly different from the control at p < 0.001.

At birth (day 0), pup body weight was significantly lower (p < 0.0001; F = 11.84) in the 800 mg/kg CaC group compared to the control and 200 mg/kg CaC groups. Body weight was not significantly different between the 200 mg/kg CaC and control groups. Subsequently, no significant difference (p > 0.05) in body weights was observed among the groups until the end of the experiment (Fig. 2).

### 3.3 Evaluation of developmental behaviour

**Fig. 3:**
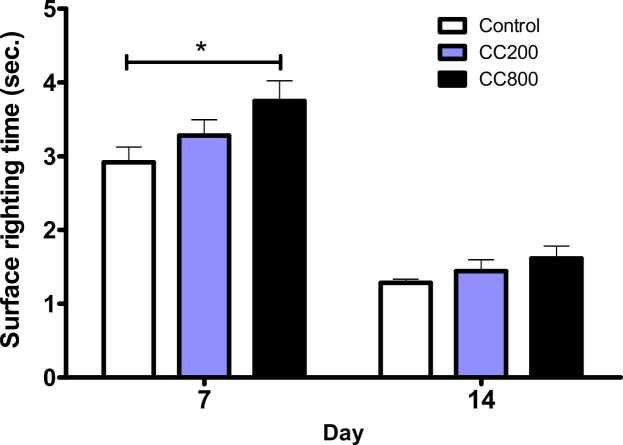
Surface right reflex activity of the pups. * - significantly different from the control at p = 0.0431. There were no other differences in the surface right reflexes, at p < 0.05. CC200 – 200 mg/kg CaC; CC800 – 800 mg/kg CaC.

On PND 7, a significantly delayed surface righting reflex time (p = 0.0431, F = 3.209) was observed in the 800 mg/kg CaC group compared to the 200 mg/kg CaC and control groups. No significant difference (p > 0.05) in surface righting reflex time was observed between the 200 mg/kg CaC and control groups. On PND 14, there were fewer surface righting reflex time among groups, although not significantly different (p = 0.2336, F = 1.474) compared to controls (Fig. 3).

**Fig. 4:**
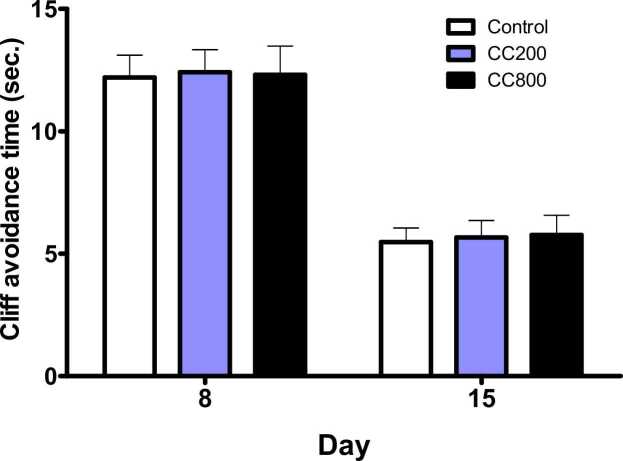
Cliff avoidance activity of the pups. There were no differences in the cliff avoidance, at p < 0.05. CC200 – 200 mg/kg CaC; CC800 – 800 mg/kg CaC.

**Fig. 5:**
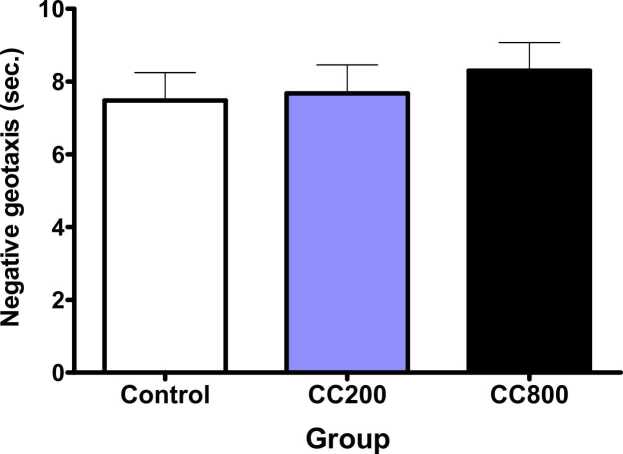
Negative geotaxis activity of the pups. There was no difference in the negative geotaxis, at p < 0.05. CC200 – 200 mg/kg CaC; CC800 – 800 mg/kg CaC.

On PND 8 and 15 (p = 0.990, F = 0.970; and p = 0.9580, F = 0.04292, respectively), the pups’ cliff avoidance time was not significantly different in the 200 mg/kg and 800 mg/kg CaC groups compared with the control, even though the avoidance time were less on day 15 (Fig. 4). On PND 10, there was no significant difference (p = 0.7021, F = 0.3549) in the negative geotaxis time in the 200 mg/kg and 800 mg/kg CaC groups compared with the control (Fig. 5). There was no significant difference in the open field test parameters (line crossing –P= 0.7167, F = 0.3350; rearing - p = 0.2662, F = 1.355; and grooming – p = 0.7167, F = 0.3350) compared with the control (Table 2).

**Table 2:** Activities of the pups in the open field maze. There were no differences in the line crossing, rearing and grooming frequencies, at p < 0.05. CC200 – 200 mg/kg CaC; CC800 – 800 mg/kg CaC.

Open field parameters	Control	200 mg/kg C. chalk	800 mg/kg C. chalk
Line crossing p = 0.1003, F = 2.394	33.85 ± 4.069	42.30 ± 4.058	30.25 ± 3.861
Rearing p = 0.2662, F= 1.355	7.350 ± 1.029	10.25 ± 1.248	9.000 ± 1.438
Grooming p = 0.7167, F = 0.3350	3.400 ± 0.7196	3.600 ± 0.4377	3.000±0.3554

### 3.4 Histology and immunohistochemistry

**Fig. 6:**
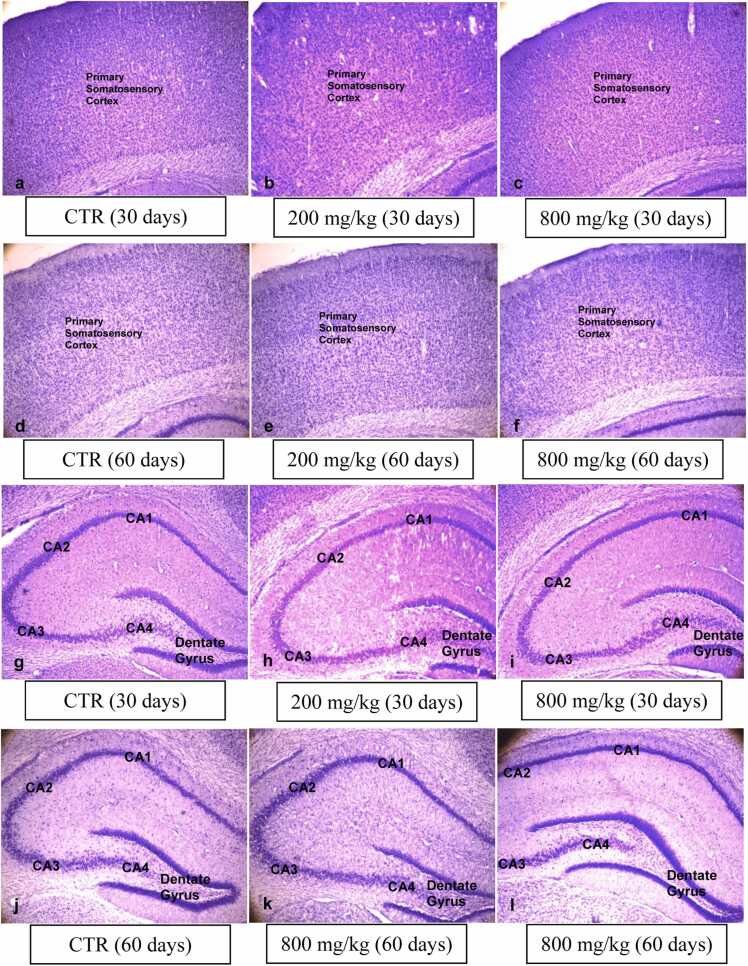
Cresyl fast violet staining for Nissl substance. The somatosensory cortex (a-f) and the hippocampus (g-l) show no adverse histological changes (4x).

In the somatosensory cortex and hippocampus, no adverse histological changes were observed in the Nissl-stained sections of the CaC groups of the 30- and 60-day-old pups compared to their matched controls (Fig. 6).

**Fig. 7:**
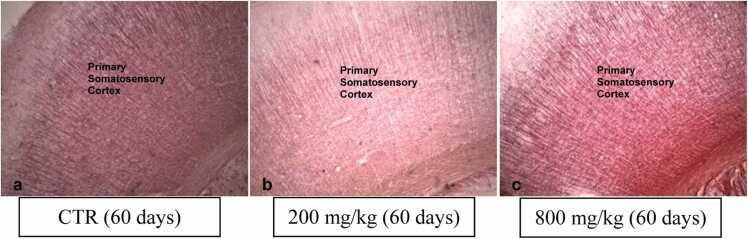
Black-Gold II staining for myelin in the somatosensory cortex (a-c) shows no hypomyelination (4x).

Black-Gold II histochemistry performed for myelin detection at the somatosensory cortex and dorsal hippocampal levels showed normal myelination in the 60-day-old pups of the CaC groups compared to control (Fig. 7).

**Fig. 8:**
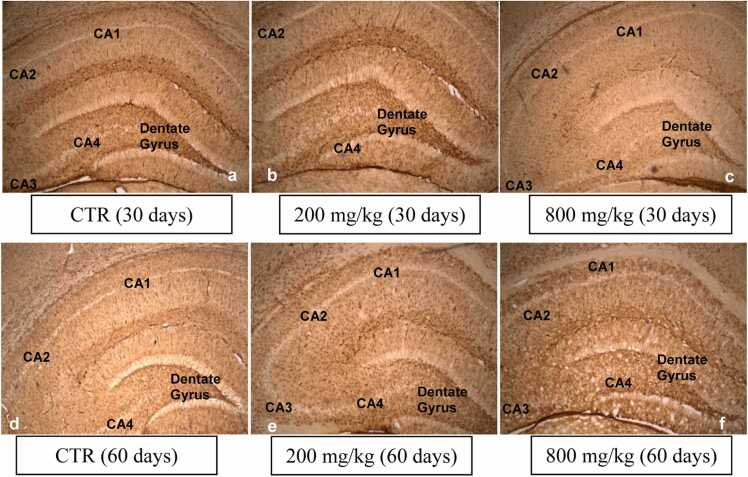
GFAP positive cells of the dorsal hippocampus (a-f). Their expression in the 30-day-old pups of the 200 mg/kg and 800 mg/kg CaC groups (a-c, 4x) and in the 60-day-old pups of 200 mg/kg and 800 mg/kg CaC groups (d-f, 4x) appear not activated.

GFAP-positive cells in the dorsal hippocampal from the 200 mg/kg and 800 mg/kg CaC groups showed no relevant signs of activation and were similar in the 30-day-old pups and 60-day-old pups compared to controls (Fig. 8).

**Fig. 9:**
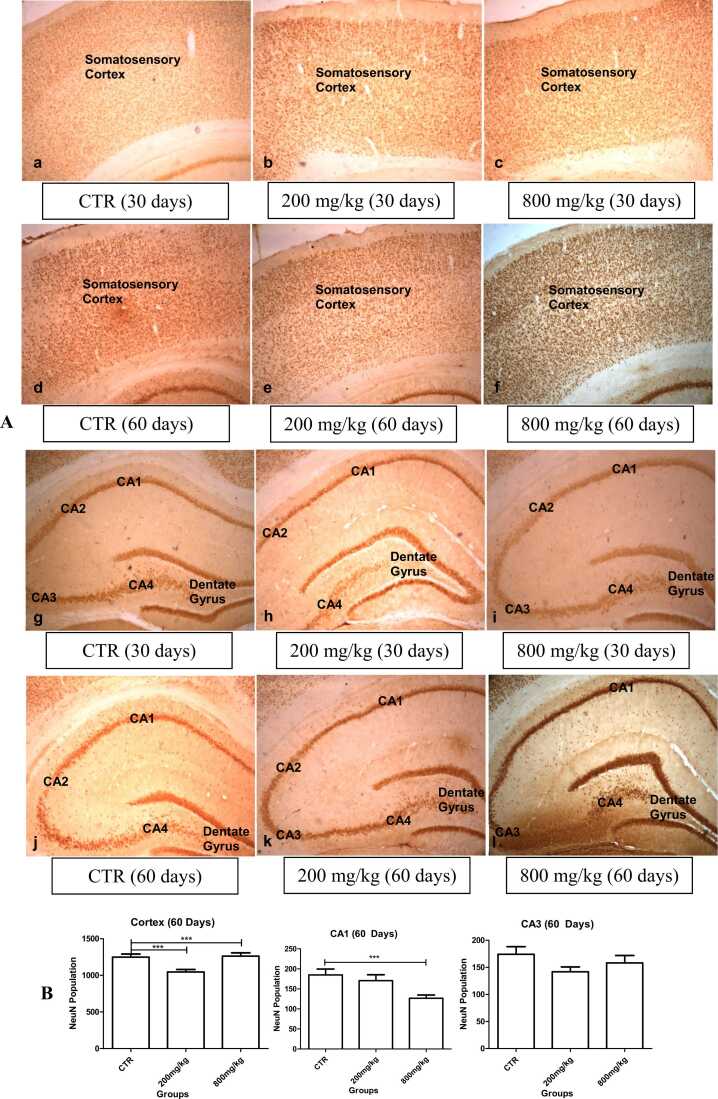
Neuronal nucleus (NeuN) immunolabelled cells. A. The somatosensory cortex (a-f) and the hippocampus (g-l) show normal neuronal integrity. B. Neurons are significantly higher in the cortex and significantly less in the CA1 of the 60-day-old in 800 mg/kg CaC group, but not different in the CA3. Neurons are significantly lower in cortex of the 60-day-old in 200 mg/kg CaC group (4x).

NeuN immunolabelled cells showed no adverse expression in the somatosensory cortex and hippocampus in the 30- and 60-day-old pups of the CaC group. In the 60-day-old pups, the neuronal population in the somatosensory cortex was significantly (p < 0.05) lower in the 200 mg/kg CaC group compared to the controls, but significantly higher in the 800 mg/kg CaC group. The neuronal population in the hippocampus was not significantly different in the CA1 and CA3 regions of the 200 mg/kg CaC group, but significantly lower in the CA1 region of the 800 mg/kg CaC group compared to the control group (Fig. 9).

**Fig. 10:**
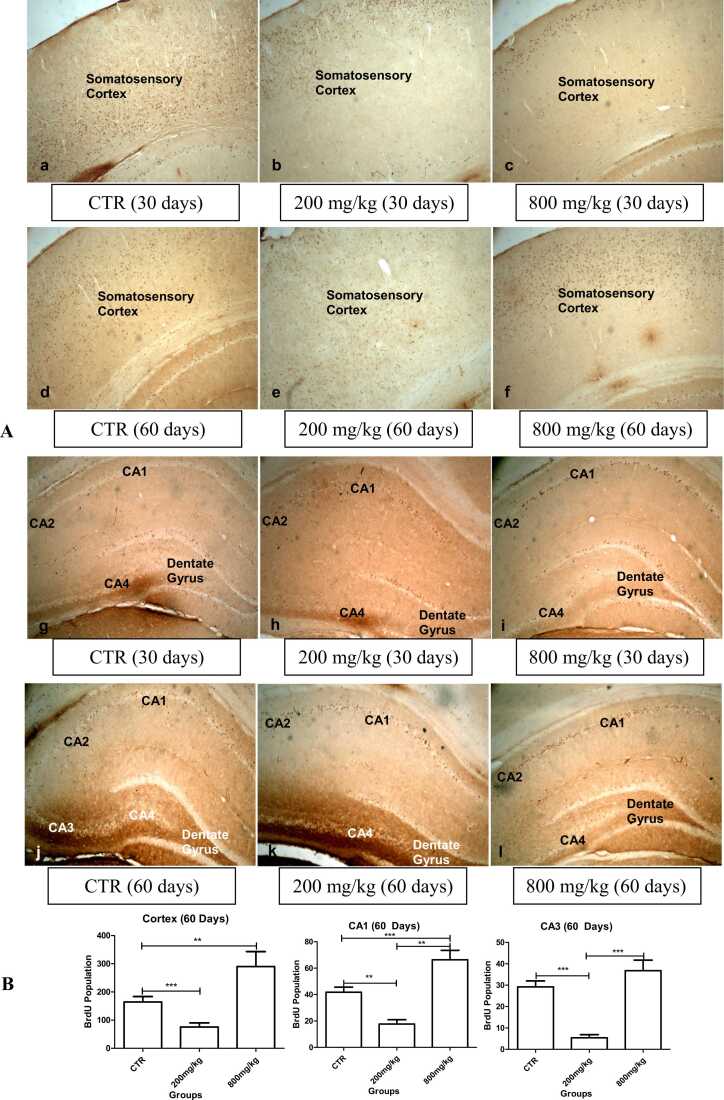
BrdU immunolabelled areas in the somatosensory cortex (a-f) and hippocampus (g-l) showing distribution of neurons in the 30- and 60-day-old rats. Neurons are significantly higher in the cortex and hippocampus of the 60-day-old pups in the 800 mg/kg CaC group, but significantly lower in the cortex and hippocampus of the 60-day-old pups in the 200 mg/kg CaC group (4x).

BrdU-positive cells were distributed in 60-day-old pups, which were significantly (p < 0.05) lower in the somatosensory cortex, CA1 and CA3 of the 200 mg/kg CaC group, but higher in the somatosensory cortex, CA1 and CA3 of the 800 mg/kg CaC group compared to their matched controls (Fig. 10).

**Fig. 11:**
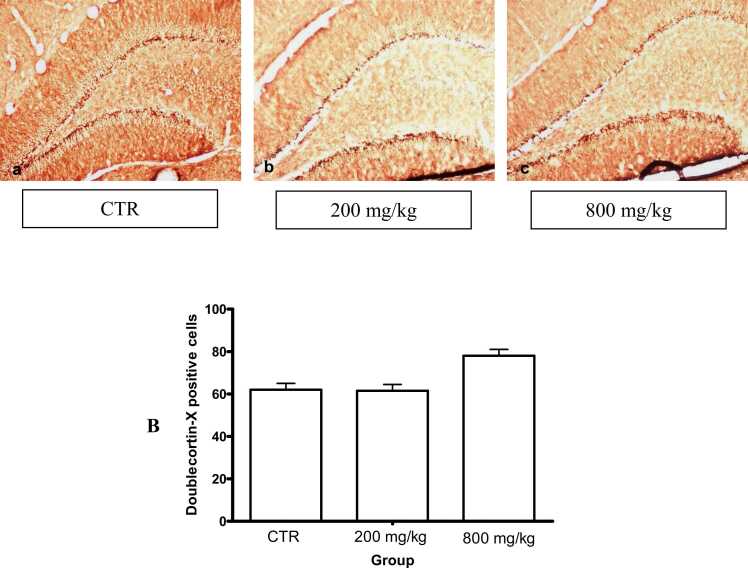
DCX immunolabelled areas of the hippocampus: A. Distribution of developing neurons in the subgranular area of the dentate gyrus of 60-day-old pups (a-c). Neurons are not different in the CaC groups (10x). B. Graphics: bar represent standard deviation.

Immunolabelling with DCX showed the distribution of developing neurons in the subgranular area in the dentate gyrus of the 60-day-old pups. Neuronal populations were not significantly different in the CaC groups compared to the controls (Fig. 11).

### 3.5 Western blot analysis

**Fig. 12:**
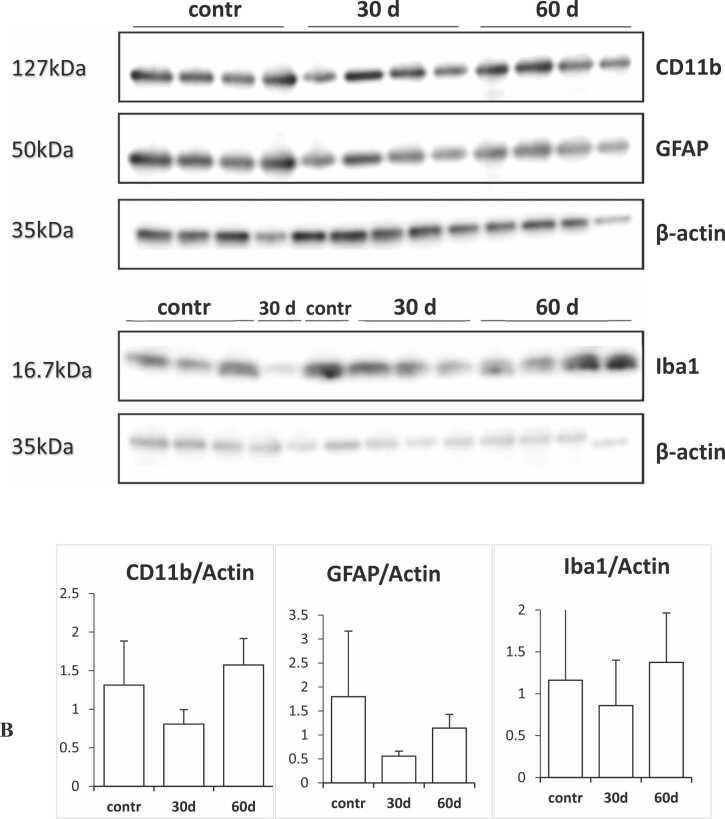
Western blotting analysis. A) Western blotting chemoluminescent bands of the samples for the three investigated markers, CD11b and Iba1 for microglia and GFAP for astrocytes. B) Graphs illustrating protein optical density normalized to the internal loading control (β-actin). No statistical differences were found among the groups. Bars represent the standard deviation.

Quantification of specific glial and astrocytic marker on control animals and pups of the 800 mg/kg group at 30 and 60 days was performed with Western Blot. Statistical analysis of the results revealed no significant differences among the groups (Fig. 12A and B).

## 4 Discussion

This research evaluated the activity of prenatal administration of CaC on the developing brain of a Wistar rat model. The results generally show that the CaC did not negatively affect the developing brain.

CaC analysis showed the presence of heavy metals, with aluminium being the highest concentration. This is unsurprising as aluminium is the core of this material (Dean et al., 2004). High concentrations of iron, potassium and calcium were observed in the CaC material, which could be beneficial where deficiency arise (Charlebois and Pantopoulos, 2023, Schiefermeier-Mach et al., 2020). However, such elements as vanadium, lead and arsenic, could be deleterious, as their concentration are beyond expected exposure levels (Balali-Mood et al., 2021, Wang et al., 2020). A comparison with previous reports showed similar trend of concentrations (DEAN et al., 2004, EKONG et al., 2015b Neelotpol et al., 2023). However, the presence of vanadium, arsenic and cadmium indicated similar regional source with Ekong et al. (2015b).

The Wistar rats in the 800 mg/kg CaC group had a lower birth weight, which subsequently did not differ over the course of the experiment from the animals of the control group. The decreased body weight may indicate disruption of nutrient absorption, possibly due to destruction of the maternal gastroesophageal lining (Ekong et al., 2012c). CaC also alters blood composition (AKPANTAH et al., 2010, EKONG et al., 2012a), which may reduce nutrient bioavailability and intake by the developing feotus. This may be the case in the present study, where recovery of body weight was observed upon cessation of exposure at birth. The group administered 200 mg/kg CaC prenatally did not show any difference in birth weight compared with the control, indicating that the dosage may not have adversely affected nutrient intake and, therefore, weight body of the developing foetus. This result is similar to that of Ekanem et al. (2015), and Ekong et al. (2015a).

Developmental cognitive and motor activities were assessed with the surface righting, cliff avoidance, negative geotaxis and open field tests. The surface righting reflex assesses the vestibular and motor systems, which control balance (FEATHER-SCHUSSLER and FERGUSON, 2016, HEYSER, 2004). There was an initial significantly delayed surface righting reflex in the 800 mg/kg, but not the 200 mg/kg CaC group, and the subsequent test was not different compared to the control. These results indicate that CaC at this dosage may have altered the vestibular and motor systems of the 800 mg/kg CaC group, the effect of which declined due to cessation of exposure and as the rats matured.

There was no difference in the cliff avoidance, negative geotaxis, and open field test parameters in the 200 and 800 mg/kg CaC group compared to matched controls, indicating that the brain areas that control these developmental processes may not have been adversely affected at the time of testing due to cessation of exposure. Cliff avoidance, negative geotaxis, and open field tests assess vestibular and motor coordination as well as anxiety (Feather-Schussler and Ferguson, 2016), and brain areas controlling these functions may not have been altered.

Histologically, the Nissl-stained somatosensory cortex and hippocampus showed no apparent adverse features in the CaC groups of 30- and 60-day-old pups compared to their matched controls, indicating that CaC at the given doses did not cause adverse brain morphological changes. Adverse changes are usually indicated by loss of Nissl granules and chromatolysis (Moon, 2018). These were not observed in the present study, which contrast to Ekong et al., (2015a), who reported an adverse histological appearance of the foetal brain. This difference may be due to the cessation of exposure, where the maturing brain may have recovered, unlike the foetus. The present result supports the non-adverse developmental behaviour studies.

Black-Gold II histochemistry for myelin showed normal myelination in the 60-day-old pups of the CaC groups compared to the control, indicating non-demyelination. The Black-Gold II histochemical technique is effective in the demonstrating myelin in formalin-fixed tissues, and reduced staining usually portends hypo-myelination (Schmued et al., 1997), which was not the case in the present study. Myelin is a lipid-rich sheath composed of tightly packed membranous layers, which is a product of oligodendrocytes (Kiernan & Rajakumar-Barr’s, 2014). The present result may also indicate that the structure or functions of oligodendrocytes were not disrupted.

GFAP positive cells of the dorsal hippocampus of the 200 mg/kg and 800 mg/kg CaC groups did not show apparently relevant activation in the 30-day-old and in the 60-day-old pups compared to controls. Western blotting quantification of glial cells expression confirms that rats, whose mothers consumed CaC during pregnancy, do not exhibit significant glial cell alterations in adulthood. GFAP expression indicates astrocytes presence (Sofroniew and Vinters, 2010), which protect the central nervous system (CNS) from threats, while helping to maintain it (Gee and Keller, 2005). Astrocytes become reactive in response to virtually all pathological conditions of the CNS and present morphological and functional changes (Haim et al., 2015), which may also be associated with the astrocytes population (Solis-Medina et al., 2018), resulting from the activity of astrocytes to maintain the brain. Astrocytes activation was not the case in the present study.

NeuN-positive cells showed no adverse integrity in the 30- and 60-day-old brains. However, 60-day-old NeuN-positive cells were significantly higher in the 800 mg/kg somatosensory cortex, while they were significantly lower in the hippocampal CA1 region of the 800 mg/kg CaC group. On the other hand, NeuN-positive cells were not significantly different in the hippocampus, but significantly lower in the somatosensory cortex of the 200 mg/kg CaC group compared to the control group. NeuN is a neuronal nuclear antigen and a marker of neuronal differentiation (Gusel’nikova and Korzhevskiy, 2015), making it specific for neurons. Since NeuN is mostly expressed in post-mitotic neurons and postnatal brain development is characterized by a change in brain population (Bandeira et al., 2009), the present results may indicate that CaC at 800 mg/kg stimulates neuronal maturity in the somatosensory cortex than in the hippocampus. Invariably, the 200 mg/kg CaC may not be adequate in this regard. This may be responsible for differences in the neuronal population of these brain areas. None the less, the hippocampal CA1 region is sensitive to injury, and may be responsible for the delayed neuronal maturation or their outright destruction upon CaC exposure and cessation.

BrdU-positive cells of the 60-day-old brains were significantly less in the somatosensory cortex, CA1 and CA3 of the 200 mg/kg CaC group, but more numerous in the 800 mg/kg CaC group compared to controls. BrdU is a marker for cell proliferation, migration, and time of origin in the CNS regardless of being neuronal or glia (DEL RIO and SORIANO, 1989, MILLER and NOWAKOWSKI, 1988). Therefore, it is useful for studying neuronal development processes, which in this case was the survival period of the labelled cells. A lower population of BrdU-positive cells in the 200 mg/kg CaC may be due to the inadequacy of the chalk at this given dose to promote maturation, leading to less survival, while the higher population in the 800 mg/kg CaC may be due to a higher survival rate, as also observed in the NeuN-positive cells. However, how this may have been achieved is elusive.

Doublecortin-X labels new neurons, as well as their dendrites (Rao and Shetty, 2004), indicating adult neurogenesis (Bordiuk et al., 2014). The DCX-positive cell population was not significantly different in the CaC groups compared to the control, indicating normal adult neurogenesis. Adult neurogenesis is hindered under adverse conditions (NANINCK et al., 2015, TAMURA et al., 2011), which was not the case in the present study.

## 5 Conclusion

In general, prenatal exposure to CaC at 200 and 800 mg/kg may not be deleterious to the developing brain, especially after exposure ceases. This is because the observed adverse actions on birth weight and surface righting reflex were reversed upon cessation of CaC exposure and maturation of the animals. Thus, this research showed normal histological and immunohistochemical values in the somatosensory cortex and hippocampus. However, the differences in the neuronal populations may be due to their ability to mature and survive after CaC exposure. The present result did not show substantial health benefit of CaC consumption, and so should be discouraged.

## Funding

This research was supported by the Universities of Uyo; the Society for Neurochemistry through the Committee for Aid and Education in Neurochemistry; the Internationalisation Programme 2016 - Action 3 of the 10.13039/501100007052University of Verona.

## CRediT authorship contribution statement

**MBE**: Conceptualization; Data curation; Formal analysis; Funding acquisition; Investigation; Methodology; Project administration; Resources; Supervision; Validation; Visualization; Roles/Writing -original draft; and Writing - review & editing. **AA**: Data curation; Formal analysis; Investigation; Methodology; Supervision; Validation; Visualization; Roles/Writing -original draft; Writing - review & editing. **IEI**: Investigation; Methodology; Project administration. **EII**: Investigation; Methodology; Project administration. **SED**: Investigation; Methodology; Project administration. **IS**: Data curation; Formal analysis; Investigation; Methodology; Supervision; Validation; Roles/Writing -original draft; Writing - review & editing. **PFF**: Resources; Supervision; Validation; review & editing. **GB**: Resources; Supervision; Validation; review & editing **MB**: Conceptualization; Funding acquisition; Resources; Supervision; Roles/Writing -original draft.

## Conflicts of Interest

Authors have no competing interest to declare.
